# 4,4′-Dimethyl-2,2′-{[2,3,3a,4,5,6,7,7a-octa­hydro-1*H*-benzimidazole-1,3-di­yl]bis­(methyl­ene)}diphenol

**DOI:** 10.1107/S1600536813015237

**Published:** 2013-06-08

**Authors:** Augusto Rivera, Héctor Jairo Osorio, Mauricio Maldonado, Jaime Ríos-Motta, Michael Bolte

**Affiliations:** a Universidad Nacional de Colombia, Sede Bogotá, Facultad de Ciencias, Departamento de Química, Cra 30 No. 45-03, Bogotá, Código Postal 111321, Colombia; b Universidad Nacional de Colombia, Sede Manizales, Colombia; c Institut für Anorganische Chemie, J. W. Goethe-Universität Frankfurt, Max-von-Laue-Str. 7, 60438 Frankfurt/Main, Germany

## Abstract

The asymmetric unit of the title compound, C_23_H_30_N_2_O_2_, contains one half-mol­ecule, with a twofold axis splitting the mol­ecule in two identical halves. The structure of the racemic mixture has been reported previously [Rivera *et al.* (2009 ▶) *J. Chem. Crystallogr*. **39**, 827–830] but the enanti­omer reported here crystallized in the *ortho­rhom­bic* space group *P*2_1_2_1_2 (*Z* = 2), whereas the racemate occurs in the *triclinic* space group *P*-1 (*Z* = 2). The observed mol­ecular conformation is stabilized by two intra­molecular O—H⋯N hydrogen bonds, which generate rings with graph-set motif S(6). In the crystal, mol­ecules are linked *via* non-classical C—H⋯O inter­actions, which stack the mol­ecules along the *b* axis.

## Related literature

For the structure of the original racemate, see: Rivera *et al.* (2009 ▶). For the use of 1,3-di­aza­heterocyclic-bridged bis­(phenols) in coordination chemistry, see: Kober *et al.* (2012 ▶); Xu *et al.* (2007 ▶). For the synthesis of the precursor, (2*R*,7*R*)- 1,8,10,12-tetra­aza­tetra­cyclo­[8.3.11^8,12^.0^2,7^]penta­decane, see: Rivera *et al.* (2012 ▶). For bond-length data, see: Allen *et al.* (1987 ▶). For graph-set analysis, see: Bernstein *et al.* (1995 ▶).
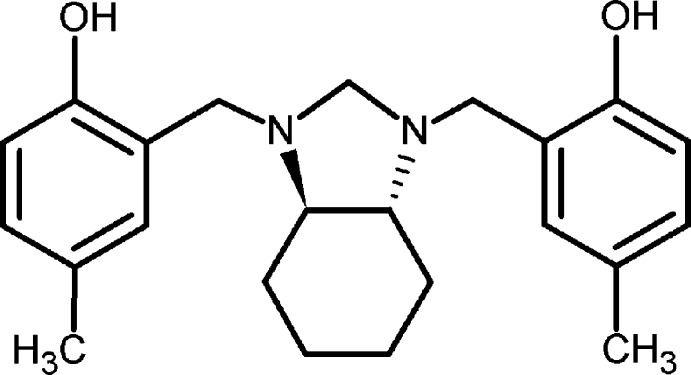


## Experimental

### 

#### Crystal data

C_23_H_30_N_2_O_2_
*M* *_r_* = 366.49
Orthorhombic, 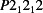

*a* = 18.5417 (9) Å
*b* = 6.0597 (4) Å
*c* = 8.9415 (5) Å
*V* = 1004.64 (10) Å^3^
*Z* = 2
Mo *K*α radiation
μ = 0.08 mm^−1^
*T* = 173 K
0.31 × 0.27 × 0.12 mm

#### Data collection

STOE IPDS II two-circle-diffractometer
Absorption correction: multi-scan (*X-AREA*; Stoe & Cie, 2001 ▶) *T*_min_ = 0.976, *T*_max_ = 0.991
12723 measured reflections
2168 independent reflections
2058 reflections with *I* > 2σ(*I*)
*R*_int_ = 0.053

#### Refinement

*R*[*F* ^2^ > 2σ(*F* ^2^)] = 0.034
*wR*(*F* ^2^) = 0.090
*S* = 1.04
2168 reflections
129 parameters
H atoms treated by a mixture of independent and constrained refinement
Δρ_max_ = 0.14 e Å^−3^
Δρ_min_ = −0.13 e Å^−3^

### 

Data collection: *X-AREA* (Stoe & Cie, 2001 ▶); cell refinement: *X-AREA*; data reduction: *X-RED32* (Stoe & Cie, 2001 ▶); program(s) used to solve structure: *SHELXS97* (Sheldrick, 2008 ▶); program(s) used to refine structure: *SHELXL2012* (Sheldrick, 2008 ▶); molecular graphics: *XP* in *SHELXTL-Plus* (Sheldrick, 2008 ▶); software used to prepare material for publication: *SHELXL2012*.

## Supplementary Material

Crystal structure: contains datablock(s) I, New_Global_Publ_Block. DOI: 10.1107/S1600536813015237/sj5327sup1.cif

Structure factors: contains datablock(s) I. DOI: 10.1107/S1600536813015237/sj5327Isup2.hkl

Additional supplementary materials:  crystallographic information; 3D view; checkCIF report

## Figures and Tables

**Table 1:** Hydrogen-bond geometry (Å, °)

*D*—H⋯*A*	*D*—H	H⋯*A*	*D*⋯*A*	*D*—H⋯*A*
O1—H1⋯N1	1.03 (4)	1.73 (4)	2.667 (2)	150 (3)
C4—H4⋯O1^i^	0.99	2.63	3.3749 (13)	133
C5—H5*B*⋯O1^ii^	0.99	2.63	3.522 (2)	150

Symmetry codes: (i) 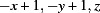; (ii) 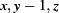.

**Fig. 1.**
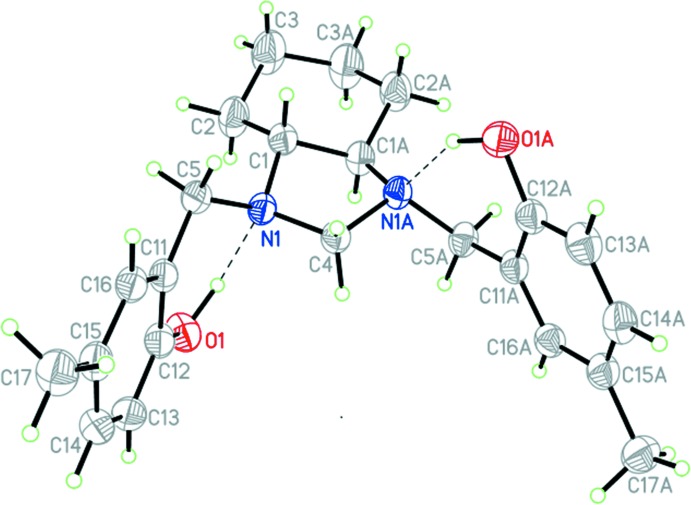
A perspective view of the title compound. Displacement ellipsoids are drawn at the 50% probability level and H atoms are shown as small spheres with arbitrary radii. Intramolecular hydrogen bonds are drawn as dashed lines.

## supplementary crystallographic information

### Comment

Chiral 1,3-diazaheterocyclic-bridged bis(phenols) form stable complexes with several metals and are of interest as ligands to metal complexes because they produce asymmetric coordination compounds (Xu *et al.*, 2007; Kober *et al.*, 2012) and therefore may be involved in enantioselective catalysis.

The title compound (I) was synthesized in a one-step reaction between the chiral macrocyclic aminal (2*R*,7*R*)-1,8,10,12-tetraazatetracyclo[8.3.11^8,12^.0^2,7^]pentadecane and *p*-cresol. Single crystals of were obtained by recrystallization from CHCl_3_ solution.

The molecular structure and atom-numbering scheme for (I) are shown in Fig. 1. The bond lengths and angles are within normal ranges (Allen *et al.*, 1987) and are comparable with those reported previously reported racemate (Rivera *et al.*, 2009). The space group, of the title enantiomer, *P*2_1_2_1_2, differs from that of the racemate which was triclinic, *P-1*. (Rivera *et al.*, 2009).

The crystal structure of (I) shows two intramolecular hydrogen bonds generating rings with graph-set motif S(6) (Bernstein *et al.*, 1995) (Table 1). In the crystal, molecules are linked *via* non-classical intermolecular C—H···O interactions, which stack the molecules along the *b* axis.

### Experimental

The title compound was synthesized according to the published procedure (Rivera *et al.*, 2012) by reacting (2*R*,7*R*)-1,8,10,12-tetraazatetracyclo [8.3.11^8,12^.0^2,7^]pentadecane (1 mmol, [α]^20^_D_ -25.1, c = O.6% CH_2_Cl_2_) and *p*-cresol (1 mmol). After work-up a solid was obtained by slow evaporation from chloroform at room temperature. After standing for two days, crystals suitable for X-ray diffraction were grown from a solution in CHCl_3_ obtained in 37% yield, m.p. = 464 K, [α]^20^_D_ -54.4, c = O.6 in CHCl_3_.

### Refinement

The hydroxyl H atom was freely refined. H atoms bound to carbon were refined using a riding model with methyl C—H = 0.98 Å, aromatic C—H = 0.95 Å, secondary C—H = 0.99 Å, and with *U*_iso_(H) = 1.5*U*_eq_(C) for methyl H or 1.2*U*_eq_(C) for aromatic and secondary H.

_reflns_Friedel_fraction is defined as the number of unique Friedel pairs measured divided by the number that would be possible theoretically, ignoring centric projections and systematic absences

### Crystal data

**Table:**

C_23_H_30_N_2_O_2_	*D*_x_ = 1.212 Mg m^−^^3^
*M**_r_* = 366.49	Mo *K*α radiation, λ = 0.71073 Å
Orthorhombic, *P*2_1_2_1_2	Cell parameters from 16515 reflections
*a* = 18.5417 (9) Å	θ = 2.2–27.5°
*b* = 6.0597 (4) Å	µ = 0.08 mm^−^^1^
*c* = 8.9415 (5) Å	*T* = 173 K
*V* = 1004.64 (10) Å^3^	Plate, colourless
*Z* = 2	0.31 × 0.27 × 0.12 mm
*F*(000) = 396	

### Data collection

**Table:**

STOE IPDS II two-circle-diffractometer	2058 reflections with *I* > 2σ(*I*)
Radiation source: Genix 3D IµS microfocus X-ray source	*R*_int_ = 0.053
ω scans	θ_max_ = 26.9°, θ_min_ = 2.2°
Absorption correction: multi-scan (*X-AREA*; Stoe & Cie, 2001)	*h* = −23→23
*T*_min_ = 0.976, *T*_max_ = 0.991	*k* = −7→7
12723 measured reflections	*l* = −11→11
2168 independent reflections	

### Refinement

**Table:**

Refinement on *F*^2^	Hydrogen site location: mixed
Least-squares matrix: full	H atoms treated by a mixture of independent and constrained refinement
*R*[*F*^2^ > 2σ(*F*^2^)] = 0.034	*w* = 1/[σ^2^(*F*_o_^2^) + (0.0482*P*)^2^ + 0.1419*P*] where *P* = (*F*_o_^2^ + 2*F*_c_^2^)/3
*wR*(*F*^2^) = 0.090	(Δ/σ)_max_ < 0.001
*S* = 1.04	Δρ_max_ = 0.14 e Å^−^^3^
2168 reflections	Δρ_min_ = −0.13 e Å^−^^3^
129 parameters	Extinction correction: *SHELXL*, Fc^*^=kFc[1+0.001xFc^2^λ^3^/sin(2θ)]^-1/4^
0 restraints	Extinction coefficient: 0.075 (11)

### Special details

**Table:**

Geometry. All e.s.d.'s (except the e.s.d. in the dihedral angle between two l.s. planes) are estimated using the full covariance matrix. The cell e.s.d.'s are taken into account individually in the estimation of e.s.d.'s in distances, angles and torsion angles; correlations between e.s.d.'s in cell parameters are only used when they are defined by crystal symmetry. An approximate (isotropic) treatment of cell e.s.d.'s is used for estimating e.s.d.'s involving l.s. planes.

### Fractional atomic coordinates and isotropic or equivalent isotropic displacement parameters (Å^2^)

**Table:**

	*x*	*y*	*z*	*U*_iso_*/*U*_eq_	
O1	0.41870 (7)	0.5018 (2)	0.60166 (17)	0.0414 (3)	
H1	0.4384 (19)	0.376 (6)	0.668 (4)	0.097 (11)*	
N1	0.44460 (7)	0.0979 (2)	0.70721 (16)	0.0303 (3)	
C1	0.45928 (9)	0.0063 (3)	0.85614 (19)	0.0340 (4)	
H1A	0.4393	−0.1468	0.8611	0.041*	
C2	0.43253 (11)	0.1352 (4)	0.9905 (2)	0.0477 (5)	
H2A	0.3792	0.1414	0.9901	0.057*	
H2B	0.4513	0.2880	0.9873	0.057*	
C3	0.45917 (12)	0.0182 (5)	1.1318 (2)	0.0596 (7)	
H3A	0.4458	0.1074	1.2204	0.071*	
H3B	0.4347	−0.1264	1.1405	0.071*	
C4	0.5000	0.0000	0.6072 (3)	0.0305 (5)	
H4	0.5217	0.1150	0.5427	0.037*	
C5	0.36990 (9)	0.0576 (3)	0.6571 (2)	0.0332 (4)	
H5A	0.3362	0.1007	0.7378	0.040*	
H5B	0.3634	−0.1021	0.6380	0.040*	
C11	0.35166 (9)	0.1843 (3)	0.5173 (2)	0.0301 (4)	
C12	0.37743 (9)	0.3996 (3)	0.4958 (2)	0.0330 (4)	
C13	0.35991 (10)	0.5134 (3)	0.3660 (2)	0.0389 (4)	
H13	0.3788	0.6571	0.3496	0.047*	
C14	0.31501 (10)	0.4180 (4)	0.2604 (2)	0.0389 (4)	
H14	0.3032	0.4983	0.1724	0.047*	
C15	0.28681 (10)	0.2075 (3)	0.2801 (2)	0.0359 (4)	
C16	0.30625 (9)	0.0935 (3)	0.4097 (2)	0.0320 (4)	
H16	0.2878	−0.0511	0.4249	0.038*	
C17	0.23790 (11)	0.1007 (4)	0.1658 (2)	0.0452 (5)	
H17A	0.2278	0.2060	0.0852	0.068*	
H17B	0.1926	0.0571	0.2138	0.068*	
H17C	0.2617	−0.0300	0.1241	0.068*	

### Atomic displacement parameters (Å^2^)

**Table:**

	*U*^11^	*U*^22^	*U*^33^	*U*^12^	*U*^13^	*U*^23^
O1	0.0387 (7)	0.0295 (6)	0.0559 (8)	−0.0030 (6)	−0.0083 (6)	−0.0022 (7)
N1	0.0243 (7)	0.0345 (7)	0.0319 (7)	0.0019 (6)	0.0008 (5)	0.0008 (6)
C1	0.0298 (9)	0.0385 (8)	0.0337 (9)	0.0043 (7)	0.0003 (6)	0.0036 (8)
C2	0.0383 (10)	0.0677 (14)	0.0371 (10)	0.0140 (10)	0.0033 (9)	−0.0034 (10)
C3	0.0501 (13)	0.0951 (19)	0.0335 (10)	0.0207 (13)	0.0048 (9)	0.0018 (12)
C4	0.0262 (10)	0.0315 (11)	0.0339 (11)	0.0007 (9)	0.000	0.000
C5	0.0251 (8)	0.0346 (8)	0.0398 (9)	−0.0014 (7)	−0.0005 (7)	0.0043 (7)
C11	0.0238 (7)	0.0296 (8)	0.0369 (9)	0.0024 (6)	0.0013 (7)	0.0006 (7)
C12	0.0274 (8)	0.0301 (8)	0.0414 (9)	0.0012 (7)	0.0003 (7)	−0.0015 (8)
C13	0.0371 (9)	0.0316 (8)	0.0482 (10)	0.0008 (7)	0.0058 (8)	0.0062 (8)
C14	0.0377 (9)	0.0410 (10)	0.0380 (9)	0.0077 (8)	0.0028 (8)	0.0062 (8)
C15	0.0305 (8)	0.0409 (10)	0.0363 (9)	0.0048 (7)	0.0014 (8)	−0.0021 (8)
C16	0.0270 (8)	0.0303 (8)	0.0388 (9)	0.0001 (7)	0.0020 (7)	−0.0013 (7)
C17	0.0427 (10)	0.0546 (11)	0.0384 (10)	0.0024 (9)	−0.0037 (8)	−0.0041 (10)

### Geometric parameters (Å, º)

**Table:**

O1—C12	1.365 (2)	C5—C11	1.505 (2)
O1—H1	1.03 (4)	C5—H5A	0.9900
N1—C1	1.468 (2)	C5—H5B	0.9900
N1—C5	1.476 (2)	C11—C16	1.392 (3)
N1—C4	1.485 (2)	C11—C12	1.403 (2)
C1—C1^i^	1.512 (3)	C12—C13	1.389 (3)
C1—C2	1.516 (3)	C13—C14	1.386 (3)
C1—H1A	1.0000	C13—H13	0.9500
C2—C3	1.531 (3)	C14—C15	1.390 (3)
C2—H2A	0.9900	C14—H14	0.9500
C2—H2B	0.9900	C15—C16	1.396 (3)
C3—C3^i^	1.530 (4)	C15—C17	1.512 (3)
C3—H3A	0.9900	C16—H16	0.9500
C3—H3B	0.9900	C17—H17A	0.9800
C4—N1^i^	1.485 (2)	C17—H17B	0.9800
C4—H4	0.9900	C17—H17C	0.9800
			
C12—O1—H1	105 (2)	N1—C5—H5B	109.2
C1—N1—C5	112.75 (14)	C11—C5—H5B	109.2
C1—N1—C4	105.47 (13)	H5A—C5—H5B	107.9
C5—N1—C4	113.58 (14)	C16—C11—C12	118.61 (17)
N1—C1—C1^i^	101.79 (11)	C16—C11—C5	120.55 (15)
N1—C1—C2	117.60 (15)	C12—C11—C5	120.78 (16)
C1^i^—C1—C2	110.66 (14)	O1—C12—C13	119.01 (16)
N1—C1—H1A	108.8	O1—C12—C11	121.19 (17)
C1^i^—C1—H1A	108.8	C13—C12—C11	119.79 (17)
C2—C1—H1A	108.8	C14—C13—C12	120.22 (17)
C1—C2—C3	108.07 (17)	C14—C13—H13	119.9
C1—C2—H2A	110.1	C12—C13—H13	119.9
C3—C2—H2A	110.1	C13—C14—C15	121.48 (18)
C1—C2—H2B	110.1	C13—C14—H14	119.3
C3—C2—H2B	110.1	C15—C14—H14	119.3
H2A—C2—H2B	108.4	C14—C15—C16	117.54 (18)
C3^i^—C3—C2	112.71 (18)	C14—C15—C17	122.16 (19)
C3^i^—C3—H3A	109.0	C16—C15—C17	120.28 (18)
C2—C3—H3A	109.0	C11—C16—C15	122.30 (17)
C3^i^—C3—H3B	109.0	C11—C16—H16	118.8
C2—C3—H3B	109.0	C15—C16—H16	118.8
H3A—C3—H3B	107.8	C15—C17—H17A	109.5
N1^i^—C4—N1	106.00 (19)	C15—C17—H17B	109.5
N1^i^—C4—H4	110.5	H17A—C17—H17B	109.5
N1—C4—H4	110.5	C15—C17—H17C	109.5
N1—C5—C11	112.24 (14)	H17A—C17—H17C	109.5
N1—C5—H5A	109.2	H17B—C17—H17C	109.5
C11—C5—H5A	109.2		
			
C5—N1—C1—C1^i^	160.26 (17)	C16—C11—C12—O1	176.16 (15)
C4—N1—C1—C1^i^	35.78 (19)	C5—C11—C12—O1	−1.1 (2)
C5—N1—C1—C2	−78.7 (2)	C16—C11—C12—C13	−2.7 (2)
C4—N1—C1—C2	156.83 (15)	C5—C11—C12—C13	180.00 (15)
N1—C1—C2—C3	−175.55 (19)	O1—C12—C13—C14	−176.61 (17)
C1^i^—C1—C2—C3	−59.2 (2)	C11—C12—C13—C14	2.3 (3)
C1—C2—C3—C3^i^	53.8 (3)	C12—C13—C14—C15	−0.4 (3)
C1—N1—C4—N1^i^	−14.09 (8)	C13—C14—C15—C16	−0.9 (3)
C5—N1—C4—N1^i^	−138.05 (15)	C13—C14—C15—C17	−179.83 (18)
C1—N1—C5—C11	170.22 (14)	C12—C11—C16—C15	1.4 (3)
C4—N1—C5—C11	−69.88 (17)	C5—C11—C16—C15	178.62 (16)
N1—C5—C11—C16	144.70 (16)	C14—C15—C16—C11	0.5 (3)
N1—C5—C11—C12	−38.1 (2)	C17—C15—C16—C11	179.38 (17)

Symmetry code: (i) −*x*+1, −*y*, *z*.

### Hydrogen-bond geometry (Å, º)

**Table:**

*D*—H···*A*	*D*—H	H···*A*	*D*···*A*	*D*—H···*A*
O1—H1···N1	1.03 (4)	1.73 (4)	2.667 (2)	150 (3)
C4—H4···O1^ii^	0.99	2.63	3.3749 (13)	133
C5—H5*B*···O1^iii^	0.99	2.63	3.522 (2)	150

Symmetry codes: (ii) −*x*+1, −*y*+1, *z*; (iii) *x*, *y*−1, *z*.
